# Computer vision models enable mixed linear modeling to predict arbuscular mycorrhizal fungal colonization using fungal morphology

**DOI:** 10.1038/s41598-024-61181-5

**Published:** 2024-05-13

**Authors:** Shufan Zhang, Yue Wu, Michael Skaro, Jia-Hwei Cheong, Amanda Bouffier-Landrum, Isaac Torrres, Yinping Guo, Lauren Stupp, Brooke Lincoln, Anna Prestel, Camryn Felt, Sedona Spann, Abhyuday Mandal, Nancy Johnson, Jonathan Arnold

**Affiliations:** 1 Institute of Bioinformatics, University of Georgia, Athens, GA USA; 2 Genetics Department, University of Georgia, Athens, GA USA; 3 School of Earth and Sustainability and Department of Biological Sciences, North Arizona University, Flagstaff, AZ USA; 4 Statistics Department, University of Georgia, Athens, GA USA

**Keywords:** Computer vision, Mask R-CNN, Arbuscular mycorrhizal fungi, Mixed linear models, Biological techniques, Computational biology and bioinformatics, Ecology, Microbiology, Plant sciences, Systems biology

## Abstract

The presence of Arbuscular Mycorrhizal Fungi (AMF) in vascular land plant roots is one of the most ancient of symbioses supporting nitrogen and phosphorus exchange for photosynthetically derived carbon. Here we provide a multi-scale modeling approach to predict AMF colonization of a worldwide crop from a Recombinant Inbred Line (RIL) population derived from *Sorghum bicolor* and *S. propinquum*. The high-throughput phenotyping methods of fungal structures here rely on a Mask Region-based Convolutional Neural Network (Mask R-CNN) in computer vision for pixel-wise fungal structure segmentations and mixed linear models to explore the relations of AMF colonization, root niche, and fungal structure allocation. Models proposed capture over 95% of the variation in AMF colonization as a function of root niche and relative abundance of fungal structures in each plant. Arbuscule allocation is a significant predictor of AMF colonization among sibling plants. Arbuscules and extraradical hyphae implicated in nutrient exchange predict highest AMF colonization in the top root section. Our work demonstrates that deep learning can be used by the community for the high-throughput phenotyping of AMF in plant roots. Mixed linear modeling provides a framework for testing hypotheses about AMF colonization phenotypes as a function of root niche and fungal structure allocations.

## Introduction

Most vascular land plants have lived in symbiotic association with Arbuscular Mycorrhizal Fungi (AMF) for more than 400 million years^1^. The plant provides carbon (C), and in return the AMF provide Nitrogen (N) and Phosphorus (P). This exchange of nutrients is central to tree diversity in forests worldwide^2^, determination of CO_2_ in the atmosphere^3^, and plant tolerance to drought, heat and pathogens^4–6^. The development of the AMF symbiosis is initiated by a single fungal hypha contacting a neighboring host root^7,8^. Insertion of the epidermal layer by the prepenetration apparatus^9^ is followed by intraradical hyphal growth. On reaching the inner cortex, branches arising from the intraradical hyphae could penetrate the cortical cell walls and form arbuscules known as the structure for nutrient transfer between symbionts^10^. Post-penetration development includes the differentiation of vesicles^11^ and spores^12^. Vesicles are nutrient storing structures for lipids and carbohydrates obtained from the plant host. AMF reproduce asexually using spores. The extensive extraradical hyphal network uptakes nitrogen, phosphorus, and other ions in soil that were otherwise inaccessible to the plant host. The various types of mycorrhizal structures differentiate from one continuum of fungal hyphae^13^ and can occur simultaneously in plant roots^14^. Seminal work has shown that hyphal length, as well as spore counts and density, can vary significantly among conspecific AMF isolates, and that this variation has been shown to be correlated with differences in plant growth^15^.

The internal development of the fungus is influenced by the plant genome^16–18^. An AMF species shows different morphological growth patterns, Arum- vs Paris-types, depending on the species of the plant partner in the association^19–21^. Large variation in AMF richness and abundance have been characterized in several plant populations, in an effort to identify symbiosis-associated genes^16,17,22–26^. Plant mutants were generated for biological validation of symbiosis genes^27^. DELLA proteins were revealed as master regulators that interact with the symbiosis signaling pathway, which provides a mechanism to integrate symbiosis with plant growth and development^28,29^. For example, DELLA transcription and protein stabilization serves to restrain plant growth but to promote arbuscule development^30,31^. Direct evidence from the greenhouse highlighted that the functioning of colonization depends not only on the plant genotype but also on the identity of AMF genera/species/isolates^23^. The relative allocation to selfish versus non-selfish fungal structures^32^ also depends on the abiotic environmental conditions. Fertilization often reduces allocation to extraradical hyphae and arbuscules relative to other structures^32^. The genotypes of the organisms involved and the environmental conditions under which they interact determine the functioning of mycorrhizal association along the mutualistic-parasitic continuum^33–40^. A better understanding of the factors is needed.

The AMF research community is limited by a lack of cost efficient and high-throughput imaging methods to quantitate the abundance of AMF hyphal structures in roots. In 1990, McGonigle et al. developed an unbiased approach for scoring AMF colonized root samples^41^. It is the gold standard until now, but it is laborious and demands skilled human scorers. Molecular quantification methods like AMF-specific phospholipid fatty acids (PLFA) approximate the amount of AM fungal biomass. DNA-based methods like quantitative real-time PCR (qPCR) allow quantification of specific AMF taxa in roots and soil. Amplicon sequencing allows the measurement of relative abundance of AMF taxa in root samples. A disadvantage of the PLFA- and DNA-based approaches is that they cannot measure colonization and morphology at the fungal structure level. Microscopy methods are synergistic by quantitating fungal structures and their morphology inside roots^41,42^. Imaging, however, requires human scorers and the process is laborious and repetitive. Preparation and visual examination of 1000 AMF slides with 20–30 root segments per slides takes an experienced researcher 2 months to complete. A computer vision model could potentially carry out this task in a few hours.

Machine learning has been applied to fungal image classification even with limited training data in *Neurospora crassa*^43,44^. A deep learning-based software, AMFinder, was developed to automate the process of quantifying AMF colonized root images^45^. The examples demonstrated computer vision as a powerful tool for high-throughput AMF phenotyping. Further improvements remain to quantitate the allocation to AM fungal structures and their morphological phenotypes in the roots using a newly available instance segmentation method of computer vision model.

Instance segmentation using deep learning techniques, like Mask R-CNN^46^, offers an opportunity for accurate and robust detection and per-pixel segmentation of different hyphal structures in root images. With the image analysis on the inferred segmentations, hyphal length/width, hyphal branching frequency, arbuscule length/width, vesicle size, spore size and other morphological traits can be automatically measured. These morphological traits can be correlated with various biological and physical processes of plants, such as photosynthesis, respiration, transpiration, and carbon and nutrient assimilation, which can be very useful for quantitative trait locus (QTL) mapping^16^ and Genome-wide Association Studies (GWAS)^17^ for symbiotic gene discovery.

Transfer learning is a technique that helps to transfer features learned from one dataset to another. The advancement of transfer learning benefits applications with limited annotated data. As of 2020, Mask R-CNN is one of the few deep learning architectures that can provide a generalist performance for instance image segmentation^47^. Transfer learning-based application of Mask R-CNN have been adopted rapidly for imaging-based plant phenotyping in recent years^48^.

We present a Mask R-CNN based image analysis method that provides the four previously unavailable advantages: (1) requires a minimal training data via transfer learning (2) achieves pixel level identification of multiple AM fungal structures via instance segmentation; (3) works on root samples colonized by a mixed populations of AMF in the field; (4) provides morphological measures on each category of AM fungal structure. We took the quantification and morphological measures from the image analysis to address fundamental questions about the AMF symbiosis: (1) can a mathematical model be developed to predict AMF colonization; (2) does the allocation to AM fungal structures vary between plants; (3) are there differences in the niche within the root system, where AMF structures are found?

To understand AMF symbiosis as part of largescale systems biology studies, we developed a deep learning-based image analysis method to automatically measure AMF colonization intensity and fungal structure morphologies. The mixed linear model was used to provide a framework for testing hypotheses about AMF colonization and the variation in these morphometric measures. The result is a direct connection between the fungal structures present in each root sample and fungal colonization of the roots. This connection will permit the exploration of how AMF affect plant health through allocation to their structures.

## Results

### Performance of Mask R-CNN on AMF image segmentation

**Figure 1:**
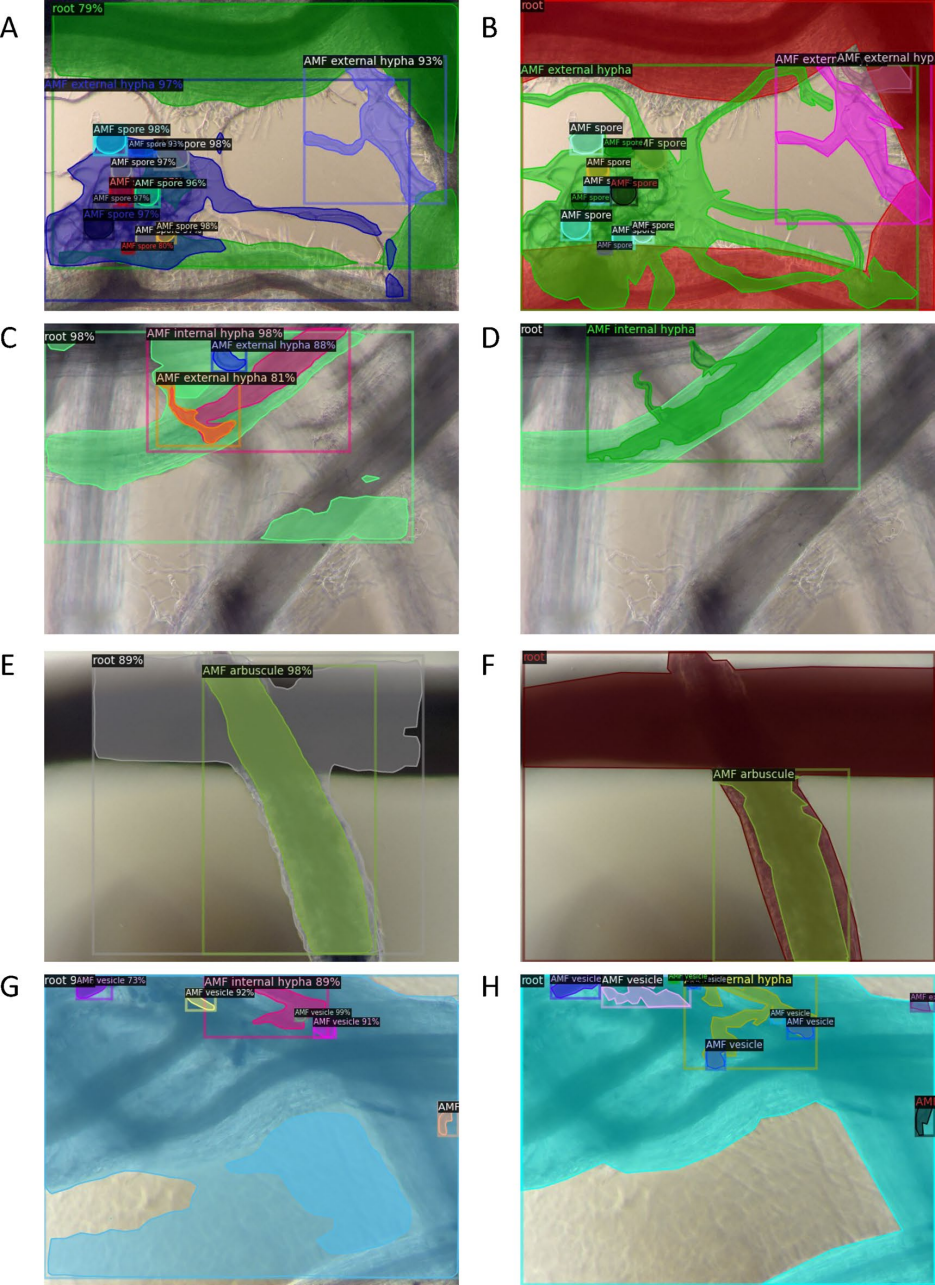
Instance segmentation on the test set from images of RIL plants grown in-house at Georgia. The left column (**A**,**C**,**E**,**G**) shows four examples of Mask R-CNN predictions of all five AM fungal structures: arbuscule, external hypha, internal hypha, vesicle, and spore. Random color is used to fill individual masks. Classification with confidence score is labeled on the corner of a bounding box. The right column (**B**,**D**,**F**,**H**) displays the ground truth annotations from human scorers.

Our Mask R-CNN model can segment AMF colonized root images with satisfying performance (Table 1). The training images and annotations were generated by human scorers using the McGonicle method^50^ on a grid associated with the 192 root intersections per slide (see “Materials and methods”). The pretrained Mask R-CNN model on the COCO dataset was loaded and trained on an in-house dataset with the default augmentation including image random flip and resize and a 0.7 confidence score threshold (Model 1) showing higher performance on our in-house testing images with 25.9 mean average precision (mAP) and 47.5 mean average precision at intersection over union (IoU) threshold of 0.5 (AP50) across classes (see “Materials and methods”). For each class, the average precision (AP) captures both the precision (related to type I error) and recall (power = 1-type II error) for IoU from 0.5 to 0.95 with a 0.05 step interval. Example results recall (power = 1-type II error) for IoU from 0.5 to 0.95 with a 0.05 step interval. Example results presented the agreement between model prediction and the ground truth (Fig. 1). The Mask R-CNN excelled at segmenting sorghum root and spore with AP values larger than 40 (Table 1). Reasonable performance was achieved on arbuscule and vesicle with AP ranging from 20 to 30. The model struggled with predicting instances of intraradical and extraradical hyphae.

**Table 1:** Performance of the Mask R-CNN compares favorably with published performance on other image datasets. The training set 1 is made of 767 in-house images on the Georgia samples. Training set 2 contains additional images from the AMFinder dataset^45,49^. The model performance is measured by average precision (AP) of each class, mean average precision (mAP) and mean average precision at intersection over union threshold of 0.5 (mAP50)^46^.

Model	Training data	Testing data	AP									mAP50
1	In-house	In-house	Root	Arbuscule	Extraradical hypha		Intraradical hypha	Vesicle		Spore	Non-AM	47.5
			46.2	29.6	6.7		7.4	21.9		55.8	13.7	
			mAP									
			25.9									
2	Combined	In-house	mAP									39.7
			21.4									
3	Combined	Combined	mAP									50.2
			29.6

**Figure 2:**
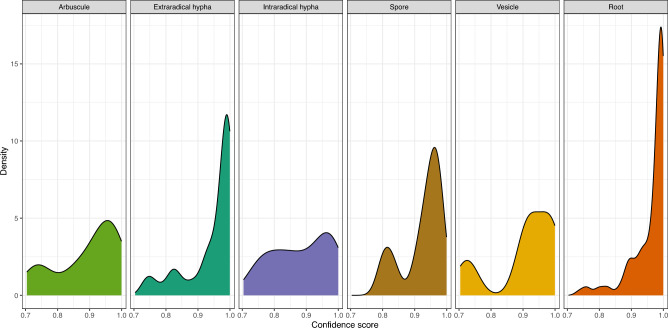
The best model trained using the in-house image dataset is used to do inference on the test set containing only in-house images of Georgia samples. Confidence scores are assigned to predicted instances during classification by the Mask R-CNN. A threshold of 0.7 was applied to select for high precision predictions only. Mask R-CNN has the highest confidence in labels assigned to predicted roots for showing a left skewed distribution.

The confidence score distributions of high precision predictions of root, extraradical hypha, vesicle and spore were left-skewed, indicating high certainty on the assigned class labels (Fig. 2). Arbuscules had lower confidence scores in comparison to sorghum roots (Fig. 2) (P < 0.00003 Tukey Multiple Comparison applied to an ANOVA of angular transformation of confidence scores). The low AP value and the high confidence score of extraradical hypha suggest that the main challenge for the Mask R-CNN is the pixel level segmentation of extraradical hypha rather than instance classification. Given the low AP value and confidence score of intraradical hypha, this fungal structure was dropped from latter analyses. Difficulty in arbuscule classification could be driven by the observation that arbuscules present in both isolation and clusters in sorghum roots. The observed frequencies of fungal structures from Mask R-CNN predictions did not differ from the frequencies counted by human scorers on the testing images (p-value = 0.786 with Fisher’s exact test). As the segmentation model produced satisfying results, we chose the best one (Model 1) for inference on a much larger in-house dataset (24,391 images) where images were collected in similar experimental settings.

The pretrained model was also trained and tested with a combined dataset including the AMFinder dataset^45^ to illustrate how the model will be iteratively improved as more data are collected. The AMFinder dataset was made up of images collected from lab grown plants with a single AMF species inoculum and had patterns different from our in-house dataset collected from plants grown in soil from the field in Georgia, USA. With learning rates 0.001 and the default augmentation, we obtained a mAP of 29.6 and mAP50 of 50.2 in the combined test set (see “Materials and methods”). This showed that our Mask R-CNN Model can be expanded and adapted to diverse conditions with different experimental settings as more representative training data are included.

We then tested whether adding the AMFinder images to the training dataset improved model performance on our in-house AMF colonized root images. The model performance was slightly decreased on the original test set. The best model trained on the combined image set had a mAP of 21.4 and mAP50 of 39.7. Whether adding new data with patterns different from the targeted conditions needs further testing with different training schedules and approaches.

### From image segmentation to measures of fungal morphology

The best performing computer vision model on in-house images presented above was applied to over 20,000 images of 108 root samples from the top, middle, bottom root regions of 12 sibling sorghum plants to generate pixel-wise segmentations of the five hyphal structures. From the segmentation results, the average quantity and size of fungal structures were computed for each root sample. Paired correlation analyses of the fungal structure morphological traits were examined first to identify whether fungal structures tend to co-occur in sorghum roots.

**Figure 3:**
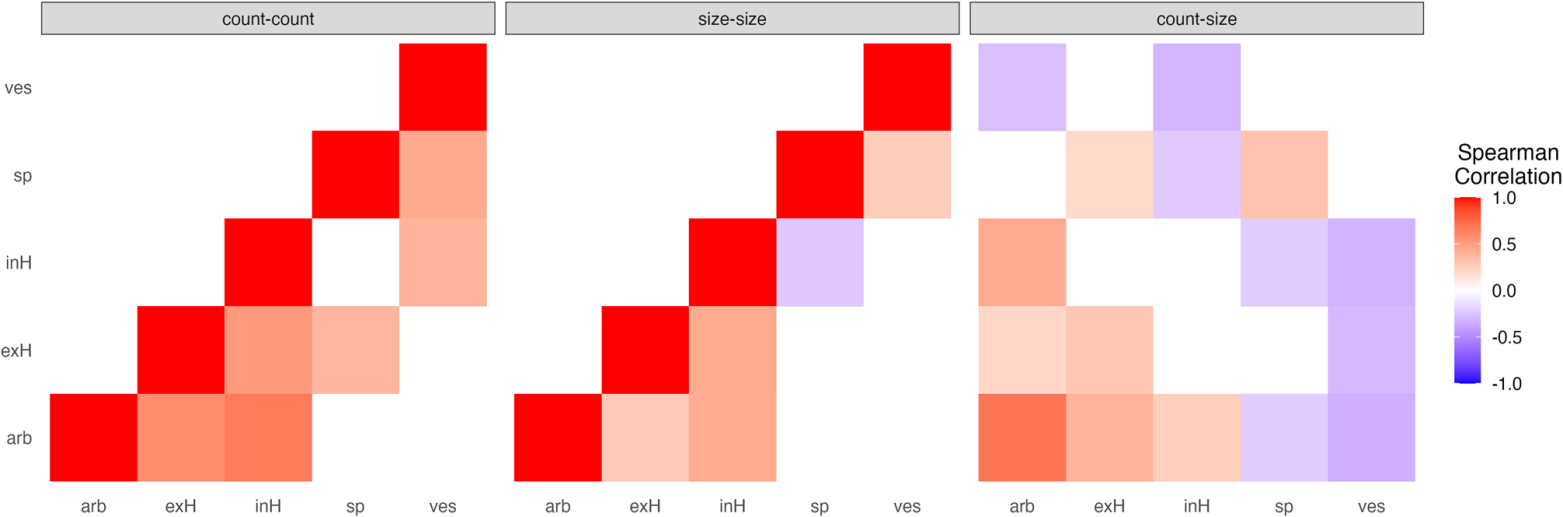
Significant correlations exist between structure counts and size. Shades of red indicate increasingly positive correlations. Shades of blue indicate decreasingly negative correlations. Fungal structures were abbreviated: arbuscule (arb), extraradical hypha (exH), intraradical hypha (inH), spores (sp), and vesicle (ves). For the correlations between fungal counts and sizes in the third panel, counts were arranged on the x-axis, sizes on the y-axis.

Positive associations dominated the frequencies of fungal structures. Higher occurrence of extraradical hyphae was consistently associated with higher occurrence of arbuscules. Vesicles and spores were positively correlated in sizes and counts. Larger number of vesicles and spores in a sample were suggestive of smaller arbuscules (Fig. 3).

### Using mixed linear models to predict total AMF abundance with fungal structure morphology

From the morphometric data of fungal structures, two measures of total AMF abundance were computed: the percentage root area occupied by AMF (percent colonization) and the density of AMF per root area (count density). Mixed linear model (MLM) analysis of the two phenotypes as response variables and the fungal structure morphology traits as predictors provides a means to test two hypotheses in the study of differential colonization by AMF. One major hypothesis is that AMF presents differential colonization between root sections. The second hypothesis is that allocation to AM fungal structures varies between plants. In this section, background on mixed linear modeling serves as an accessible introduction to how MLMs can be used to test these two hypotheses.

#### The rationale for mixed effects

Mixed Linear Models (MLMs), also known as multilevel or hierarchical models, feature fixed and random effects^51^ (Box 1). Experimental treatments are typically modeled as fixed effects. Individual observations are grouped by random factors. Random factors, therefore, constitute the grouping level. Fixed factors are estimated as the mean effect for a particular factor level. In contrast, if the primary interest lies in estimating between-group variances, variables are modelled as random effects. The estimated values of random factors are shrunk towards the population mean.

**Figure 4:**
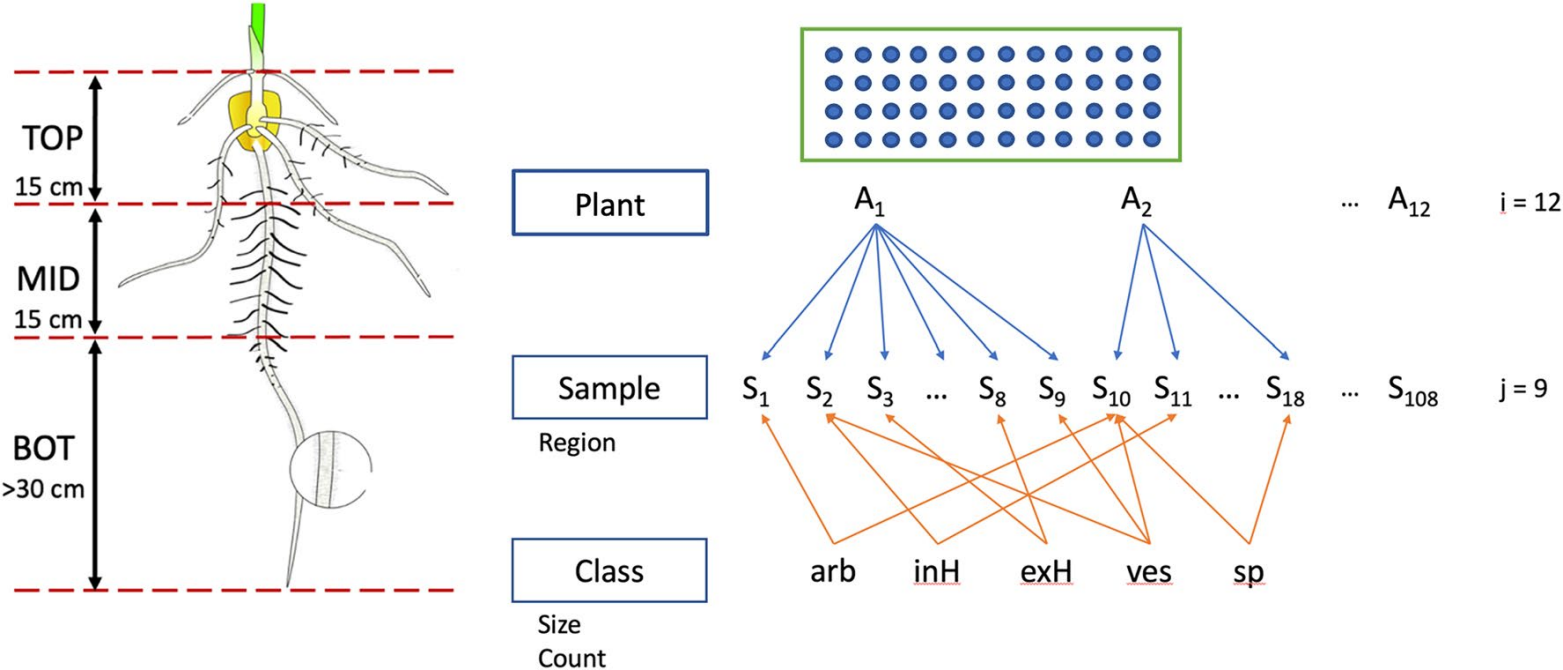
Experimental design entails hierarchical sampling first from a RIL population. Twelve inbred lines were used in the experiment. One plant of each inbred line was sampled from the top (TOP), middle (MID), and bottom (BOT) of the root system. Each section was sampled three times. For each sample, 4 fields of view were imaged around each marker dot as shown on the schematic slide below, generating 192 root intersections per slide. Five fungal structures: arbuscule (arb), internal hypha (inH), external hypha (exH), vesicles (ves), and spore (sp) were segmented from each image. Root region is a variable recorded for each sample. Average size and count are computed for each class of instances found in a sample.

The choice of using mixed effects to model AMF colonization is motivated by the experimental design. Our data are inherently hierarchical. AMF colonization was quantified in each of the three root regions within each sorghum plant, and three replicates were taken per root region (Fig. 4). The nested layers are plant, root depth, and replicate. The spatial scales between root samples and sorghum plants are biologically nested^52^. Between-sample variance needs to be evaluated as a random factor. It would be wrong to treat root samples from the same sorghum plant as independent. The twelve sorghum plants are siblings randomly sampled from a RIL population. Between-plant variance is treated as a random factor as well. Root region and AMF structure level phenotypes are the fixed effects. The same model structure can be used to model the AMF count density.

#### Mixed linear model assumptions

In MLM, both root samples and sibling plants are assumed to be randomly sampled from a population of samples and a population of sorghum recombinant inbred lines, respectively^53^. It is assumed that the sampling errors (ε_ij_) and the plant level random effects (u_0i_) are independent and that the random effects and sampling errors have a normal distribution of zero mean and distinct constant variances (Box 1). Another assumption is that the mean and variance of the response variable are not functionally dependent on each other across siblings. Transformation of the response variable is an effective method to remove the dependency. Choice of models are designed to address each of the three questions of the paper laid out at the end of the introduction.

#### The null model (Model 1)

The Null MLM Model does not include explanatory variables but only the mean overall AMF colonization and the plant level and sample level differences in colonization. The colonization by AMF of a root sample from a plant (y_ij_) is equal to the mean colonization in the sorghum population (β_0_) plus the plant level random difference from the population mean (u_0i_) plus the sample level differences (ε_ij_) (Box 1). This simple Null Model shows that MLM partitions the total variance in AMF colonization var(y_ij_) into a variance between plants var(u_0i_) and a variance between samples var(ε_ij_) as shown from Box 1 (Model 1^54^).

$$var\left({y}_{ij}\right)=var\left({u}_{0i}\right)+var\left({\epsilon }_{ij}\right)={\sigma }_{u}^{2}+{\sigma }_{\epsilon }^{2}$$

The between plant variance var(u_0i_) was estimated to be 0.0099 and two times that of the between sample variance var(ε_ij_) in Table 2. The proportion of the total percent colonization differences can be quantified at the plant level by computing the intraclass correlation (ICC)^54^.

**Table 2:** Mixed linear models are well predicted for percent colonization by arbuscule count, arbuscule size, region, and plant. Proportional change in variance (PCV) is provided to measure the importance of random effects. Intraclass correlation (ICC) is used to implicate the variance between plants. Varied measures of fit are provided to assess model performance.

	Random intercept					Random intercept + slope
	Model 1	Model 2	Model 3	Model 4	Model 5	Model 6
	Null model	Model with one fixed effect	Model A with two fixed effects	Model B with two fixed effects	Model A with three fixed effects	Model B with three fixed effects
Fixed Effects						
Intercept	0.3339	0.3360	0.3329	0.3307	0.3293	0.3410
regionTOP	NA	0.0492	0.0354	0.0312	0.0241	0.0226
regionBOT	NA	− 0.0552	− 0.0322	− 0.0213	− 0.0100	− 0.0087
arb_count_scaled	NA	NA	NA	0.0885	0.0757	0.0937
arb_size_scaled	NA	NA	0.0490	NA	0.0346	0.0304
Variance of fixed effects	NA	0.0018	0.0040	0.0092	0.0109	0.0139
Random effects						
Variance between plants	0.0099	0.0101	0.0056	0.0015	0.0010	0.0009
Variance between root samples	0.0054	0.0034	0.0024	0.0015	0.0009	0.0007
Proportional change in variance (PCV)						
Between plants	NA	− 0.0221	0.4355	0.8447	0.9003	0.9091
Between root samples	NA	0.3648	0.5640	0.7298	0.8344	0.8713
Intraclass correlation (ICC) or variance partition coefficient (VPC)						
Plants	0.6472	0.7470	0.7037	0.5133	0.5248	0.5645
Model performance						
Marginal R^**2**^	NA	0.1190	0.3345	0.7539	0.8525	0.8532
Conditional R^**2**^	0.6472	0.7771	0.8028	0.8802	0.9299	0.9574
AIC	− 217.9263	− 259.5121	− 301.6817	− 362.4751	− 413.8518	− 421.9404
BIC	− 209.8799	− 246.1014	− 285.5889	− 346.3823	− 395.0769	− 397.8012
Deviance	− 223.9263	− 269.5121	− 313.6817	− 374.4751	− 427.8518	− 439.9404

$$ICC= \frac{var\left({u}_{0i}\right)}{var\left({u}_{0i}\right)+var\left({\epsilon }_{ij}\right)} =\frac{{\sigma }_{{u}_{0}}^{2}}{{\sigma }_{{u}_{0}}^{2}+{\sigma }_{\epsilon }^{2}}$$

In Table 2, the ICC of Null Model was 0.647, which implies that 64.7% of differences in total percent colonization of AMF is at the plant level and could be controlled by the plant genome^55^. An alternative hypothesis would be that this clustering of variance at the plant level might be attributable to the different composition of AMF structures, and this composition could be defined by the plant genome. We will come back to testing the alternative hypothesis in Models 3, 4, and 5.

#### Random intercept model with experimental design variable (Model 2)

In Model 2, the Null Model (Model 1) is expanded by including the design variable, the root region where the root sample was obtained (Fig. 4), with fixed effect β_1_. Root region is a discrete predictor with three levels. The goal of the model is to investigate if AMF percent colonization differs between root regions and to determine the extent to which variance at plant level may change after taking into account differences in colonization in root regions. Proportional change in variance (PCV) at different levels can be computed to evaluate the change using the following equation^56,57^,

$${PCV}_{plant}=\frac{var\left({u}_{0i}\right)-va{r}^{\prime}\left({u}_{0i}\right)}{var\left({u}_{0i}\right)}$$

$${PCV}_{sample}=\frac{var\left({\epsilon }_{ij}\right)-va{r}^{\prime}\left({\epsilon }_{ij}\right)}{var\left({\epsilon }_{ij}\right)}$$

where var(u_0i_) is the between plant variance in the Null Model and var’(u_0i_) is the between plant variance in the new model. Comparing Model 2 to the Null Model 1, the PCV_plant_ was equal to − 0.0221, and PCV_sample_ was equal to 0.3648 in Table 2. We concluded that 36.48% of sample variance within plants in the null model is attributed to differences in root regions. By adjusting for the root regions where the sample was obtained, another 2.21% of the variance in percent colonization by AMF was accounted for by plant differences in Table 2.

#### Random intercept + fixed slope model with AMF structure predictors (Model 3, 4, 5)

As mentioned earlier under the Null Model, an alternative hypothesis to the clustering of total percent colonization of AMF at the plant level is that the plant level differences in AMF abundance can be attributable to the different composition of AMF structures in the plants, which could be controlled by the plant genome^55,58^. To test the possibility, the three Models (3, 4, and 5) expand Model 2 by including arbuscule count and/or size as fixed effects determined to be significant by Lasso Regression^59^. By comparing Models 3, 4, and 5 to the Null Model, the changes in plant level variance can be measured using ICCs and PCVs after adding different AMF structure predictors to the model (Table 2). The interpretation of ICCs and PCVs leads to a major conclusion regarding the total AMF percent colonization and the composition of fungal structures, which is discussed in depth in a later section.

The effect of average size of arbuscule (β_2_) and the effect of arbuscule count (β_3_) per sample are continuous variables that describe the association of the AMF structures with the total degree of root colonization by AMF (y_ij_)s. A positive estimate of β_3_, for example, indicates a positive linear relation between AMF colonization and average arbuscule size. A larger estimate of β_3_ than β_4_ means arbuscule size has a stronger effect on total AMF colonization per unit increase than arbuscule count. In all three models, the relations between fixed effects and overall AMF colonization are considered to be the same in all sorghum plants. In other words, the slopes are fixed with respect to plant.

#### Random intercept + random slope model with AMF structure predictors (Model 6)

In Model 6, the effect of arbuscule count (β_3_) on overall AMF colonization may differ between sorghum plants. For examples, in some plants with high AMF colonization in the roots, arbuscules may be the dominant hyphal structure but not in other plants. In Model 6, the regression coefficient of AMF colonization on arbuscule count varies at the plant level to capture this differential effect. By comparing Model 6 to Model 5, it is possible to determine whether the assumption of varying magnitude of association of arbuscule count and percent colonization between plants holds.

The total variance in AMF colonization var(y_ij_)is still made up of two parts, a variance between plants var(u_0i_, u_1i_x_1ij_) and a variance between samples var(ε_ij_). The variance between plants var(u_0i_, u_1i_x_1ij_), however, partitions into a slope variance var(u_1i_x_1ij_), intercept variance var(u_0i_) and their covariance cov(u_0i_, u_1i_x_1ij_)^60^. This variance is a quadratic function in arbuscule count:

$$\begin{aligned} var\left( {y_{ij} } \right) & = var\left( {u_{0i} ,u_{1i} x_{1ij} } \right) + var\left( {\varepsilon_{ij} } \right) \\ & = var\left( {u_{0i} } \right) + var\left( {u_{1i} x_{1ij} } \right) + 2cov\left( {u_{0i} ,u_{1i} x_{1ij} } \right) + var\left( {\varepsilon_{ij} } \right) \\ & = \sigma_{u0}^{2} + \sigma_{u1}^{2} x_{1ij}^{2} + 2\sigma_{u0u1} x_{1ij} + \sigma_{\varepsilon }^{2} \\ \end{aligned}$$

When there are random slopes in the model, the *Variance Partition Coefficient* (VPC), a function of arbuscule count, is calculated to measure the relationship of plant level variance to the total variance rather than an ICC^60^:

$$VPC= \frac{var({u}_{0i},{u}_{1i}{x}_{1ij})}{var\left({u}_{0i},{u}_{1i}{x}_{1ij}\right)+var({\epsilon }_{ij})}=\frac{{\sigma }_{{u}_{0}}^{2}+{\sigma }_{{u}_{1}}^{2}{x}_{1ij}^{2}+2{\sigma }_{{u}_{0}{u}_{1}}{x}_{1ij}}{{\sigma }_{{u}_{0}}^{2}+{\sigma }_{{u}_{1}}^{2}{x}_{1ij}^{2}+2{\sigma }_{{u}_{0}{u}_{1}}{x}_{1ij}+{\sigma }_{\epsilon }^{2}}$$

VPC is similar to ICC in terms of interpretation of the result.

Box 1

**Figure:**
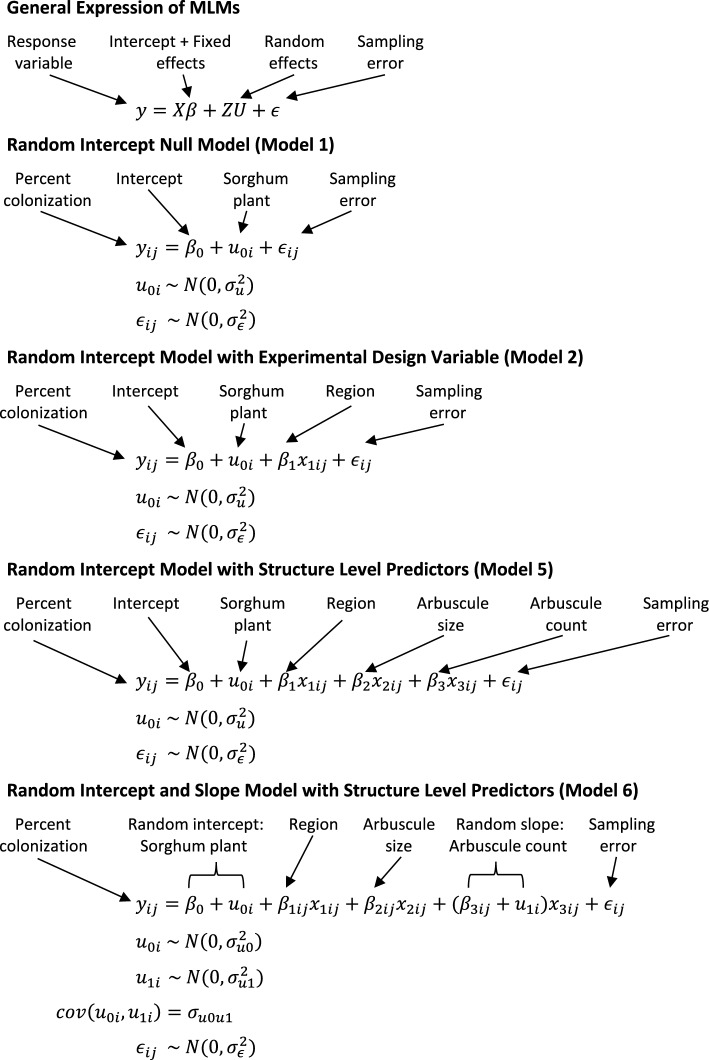
Box 1: Mixed linear models^51^ discover relations between AMF colonization and count density on the one hand and fungal morphology on the other hand

### Arbuscule morphology is predictive of differential AMF percent root colonization in sibling plants

Looking at how the plant level variance changes as predictors such as, root region, arbuscule size and count were added to the Null Model in Model 2, 3, 4, 5, we observed that, in Model 6, 90.9% total variance in percent colonization is attributed to the plants (PCV_plant_ = 0.9091). An VPC of 56.6% suggested that even if variance at plant level shrunk, it still explained the majority of differences in percent colonization. This is possible because the sample level variance dropped with the plant level variance by 87.1% as arbuscule count and size were added as predictors.

**Figure 5:**
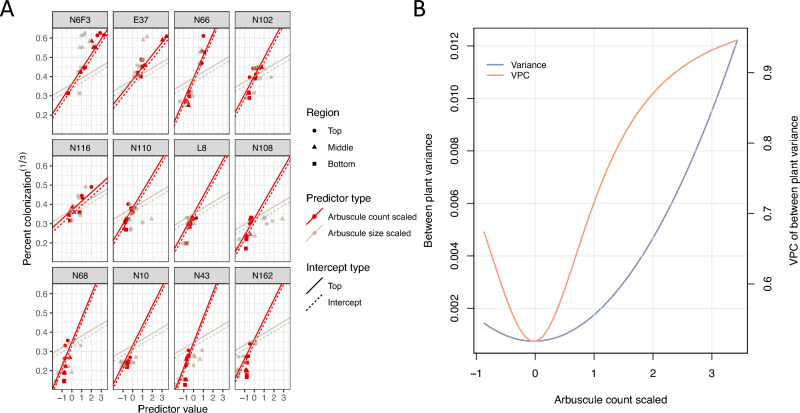
Percent colonization is well predicted by Model 6. (**A**) Random slopes (red) are fitted to scaled arbuscule count for each plant. Fixed slopes for arbuscule size (grey) are shown as reference for easy visualization of the changing slopes of arbuscule count. The solid line represents the intercept of the top root region, which predicts for higher AMF percent colonization than sample mean. (**B**) Variance between plants and its VPC are plotted as a function arbuscule count. Scaled arbuscule count is plot on the x-axis, with Variance between plants as the main y-axis, its VPC on the secondary y-axis.

Model 6 had the highest R-squared^57,61^ of 0.9574 and desired lowest information criteria and deviance. A model with random slopes for both arbuscule count and size were fitted but not included in Table 2 as it was not significantly different from Model 6 in a likelihood ratio test. Confidence scores for arbuscule was added as a fixed effect to Model 6 and was tested nonsignificant using a likelihood ratio test. In Fig. 5a, the expected values of AMF colonization from Model 6 were plotted to visualize the fit of the model to the data and the varying slopes between plants. The plant level variance and its VPC is a function of arbuscule count in Model 6 (Fig. 5b). The clustering of plant level variance was stronger when more arbuscules were observed, also reflected by the increasing of VPC (Fig. 5b). For example, N6F3, E37, N66, N102, and N116 had higher slopes and hence higher arbuscule counts than the remaining accessions. The remaining plants, however, had lower arbuscule counts and similar total AMF percent colonization. It was impossible to distinguish the remaining plants by their arbuscule counts. At first sight it seemed strange that arbuscule count should be selected as a predictor when it had lower confidence scores than other traits (Fig. 2), but as shown in Fig. 5B, there were substantial differences in arbuscule count for the first 5 accessions, and that was why arbuscule count was selected for inclusion in the mixed linear model to explain plant level differences.

#### The plant level variance component in count is stable across models: loss of goodness of fit in random effects is offset by a gain in goodness of fit to fixed effects of fungal structures

The same variable selection and model selection procedure for overall AMF percent colonization was applied to fit overall AMF count density as the response variable. The β vector of fixed effects was modified to include the appropriate AMF structure phenotypes as predictors.

Lasso regression^59^ selected the count measures of all fungal structures as fixed effects in the mixed linear model to predict overall AMF count density in sorghum plants. Forward selection removed the number of intraradical hyphae as an explanatory variable. Root regions and the counts of arbuscules, vesicles, spores and extraradical hyphae were the five fixed effects in Model 3 and 4 for count density. Random slopes were added with respect to the four effects of fungal structures. The effect of extraradical hyphae was the only one that differed between the sorghum plants. A random slope was added to the effect of extraradical hyphae in Model 4. We added the confidence score of extraradical hypha as a fixed effect to count density Model 4 and found that it was not a significant variable.

The proportion of variance at the plant level remained stable as fixed effects were added to models (Table 2). In Table 3, the ICCs and VPC of between plant variance ranged from 0.428 to 0.490, which was less than a 7% difference in variance explained. The proportional changes in plant variance decreased by 70.9%, which was compensated for by a 77.4% drop in sampling variance. What variation in count density lost to the fixed effects was replaced by the improved fit of the model.

**Table 3:** Mixed linear models are well predicted for count density by counts of hyphal structures, root regions, and sorghum accessions. Proportional change in variance (PCV) is provided to measure the importance of random effects. Intraclass correlation (ICC) is used to implicate the variance between accessions. Varied measures of fit are provided to assess model performance.

	Random intercept			Random intercept and slope
	Model 1	Model 2	Model 3	Model 4
	Null model	Model with one fixed effect	Model A with five fixed effects	Model B with five fixed effects
Fixed effects × 10^9^				
Intercept	809,770.357	793,371.277	786,651.710	790,215.150
regionTOP	NA	114,053.586	76,643.165	86,253.886
regionBOT	NA	− 64,856.345	− 7287.224	− 8284.854
arb count scaled	NA	NA	66,137.475	60,660.157
exH count scaled	NA	NA	60,918.089	63,380.104
sp count scaled	NA	NA	47,678.393	39,526.530
ves count scaled	NA	NA	97,338.416	111,460.129
Variance of fixed effects	NA	5.520	33.276	34.734
Random effects × 10^9^				
Variance between plants	20.689	21.322	7.049	6.012
Variance between root samples	27.674	21.979	7.642	6.264
Proportional change in variance (PCV)				
Between plants	NA	− 0.031	0.659	0.709
Between root samples	NA	0.206	0.724	0.774
Intraclass correlation (ICC) or variance partition coefficient (VPC)				
Plant	0.428	0.492	0.480	0.490
Model performance				
Marginal R^**2**^	NA	0.113	0.694	0.692
Conditional R^**2**^	0.428	0.550	0.841	0.875
AIC	− 1543.514	− 1563.654	− 1674.505	− 1678.541
BIC	− 1535.467	− 1550.243	− 1650.366	− 1649.037
Deviance	− 1549.514	− 1573.654	− 1692.505	− 1700.541

#### Differential AMF colonization between sorghum root regions

**Figure 6:**
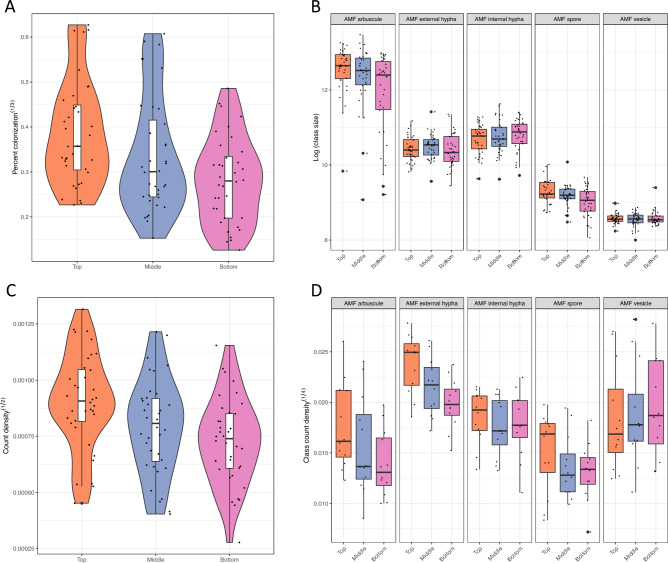
AMF colonization and structure abundance vary with root depth. (**A**) and (**B**) show the distribution of total AMF percent colonization and count density by the top, middle, bottom root regions. Colonization is the highest in the top root region. To examine the reason for high AMF colonization in the top root region, Box and Whisker plots are used to display the size and count density of each AM fungal structure by root regions in (**C**) and (**D**).

A significant improvement of Model 2 to the Null Model 1 for both total AMF colonization phenotypes supports AMF colonization to be different between root regions. PCV_sample_ was 0.365 and 0.205 respectively for AMF percent colonization and count density (Tables 2 and 3). The positive signs of PCVs suggest that the sample variances within plants in the null models are attributed to differences in root regions. The top root region had the highest colonization by t-tests at the 0.05 significance level (Fig. 6a,b). Arbuscule count was a predictor essential for the modeling of both phenotypes in the previous section. It is a reasonable speculation that arbuscule count is a main driving force in the positive correlation of the two total AMF colonization traits. The speculation is sustained by larger arbuscule size (Fig. 6c), higher numbers of arbuscules and extraradical hyphae in the top root region (Fig. 6d), tested significant using Tukey Multiple Comparison tests (Table 4).

**Table 4:** Tukey multiple comparison contrasts between region levels with their significance (p-values). The top, middle, and bottom root regions were abbreviated as TOP, MID and BOT.

Contrast	*arb*		exH		inH		sp		ves	
	Estimate	p-value	Estimate	p-value	Estimate	p-value	Estimate	p-value	Estimate	p-value
Structure count										
MID-TOP	− 0.203	0.2538	− 0.527	0.0015	− 0.109	0.7834	− 0.211	0.5379	0.192	0.4038
MID-BOT	0.383	0.0096	0.385	0.0274	− 0.193	0.4663	0.205	0.5580	− 0.018	0.9922
TOP-BOT	0.586	< 0.0001	0.912	< 0.0001	− 0.084	0.8636	0.416	0.0957	− 0.209	0.3397
Structure size										
MID-TOP	− 0.281	0.2341	0.162	0.6823	0.229	0.4966	− 0.144	0.7754	− 0.170	0.7275
MID-BOT	0.469	0.0201	0.159	0.6942	− 0.075	0.9274	0.308	0.3275	− 0.243	0.5247
TOP-BOT	0.750	< 0.0001	− 0.004	0.9998	− 0.304	0.2944	0.447	0.0922	− 0.073	0.9434

**Figure 7:**
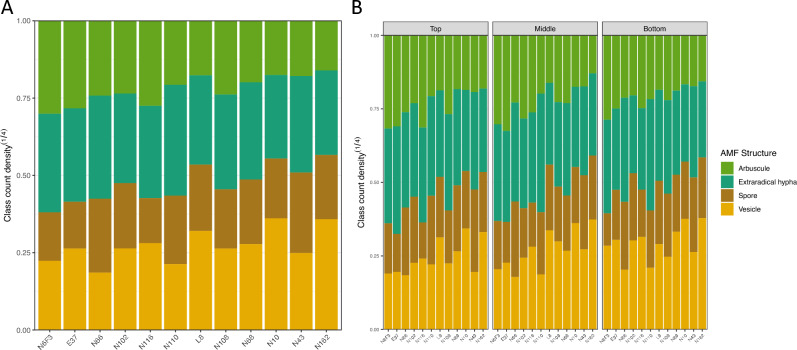
The stack bar plots reflect the relative abundance of AM fungal structures when the total AMF count density is assumed to be the same across sibling plants. Sibling plants are ordered in decreasing total AMF percent colonization. (**A**) The relative abundance of arbuscule and extraradical hypha decreases as the plant has more quantities of vesicle and spore. (**B**) The same relationship is observed in the top, middle and bottom root regions of sibling plants.

Although the morphological traits of other AM fungal structures did not have significant effects, they could still contribute to differential colonization. If the sorghum plants were colonized by equal amounts of AMF, Fig. 7a,b showed how the relative abundance of AM fungal structures in the roots could differ. When the twelve sorghum plants were ranked in decreasing order of AMF percent colonization from left to right, the relative amount of arbuscules and extraradical hyphae trended downward. The same observation held if the panel was divided by root regions.

**Figure 8:**
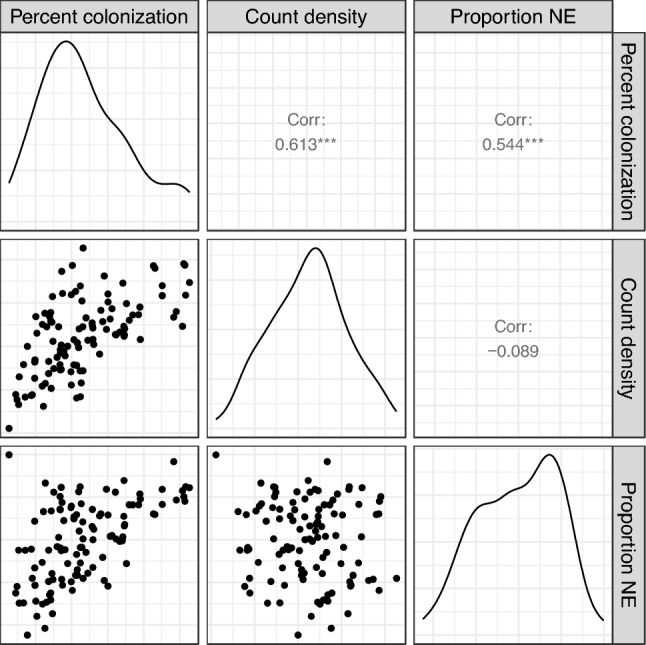
The density plots of AMF percent colonization, count density and proportion of nutrient exchange (Proportion NE) are arranged on the diagonal. The phenotypes were transformed to be normally distributed. Scatter plots of each pair of phenotypes are visualized in the lower panel. Pearson correlation values and significance are displayed in the upper panel. For example, percent colonization and proportion NE has a 0.544 positive correlation.

To quantify the observation, one more phenotype was calculated, the amount of arbuscule and extraradical hyphae divided by the total AMF structures. It measures the proportion of nutrient exchange (PNE) structures^32^. After logit transformation, a mixed linear model was fitted to PNE with root region as the fixed effect and a plant level random effect. The intraclass correlation for PNE was 0.585. The conditional R-squared of the mixed linear model was 0.624. Tukey multiple comparison test showed that the top 0–15 cm and the middle 15–30 cm tested insignificant to each other, but both were tested significant to the bottom > 30 cm roots (Table 5). Percent colonization and count density had the middle 15–30 cm root region tested insignificant against the bottom root regions. The levels of AMF colonization of the twelve sorghum plants were ranked differently with percent colonization, count density and PNE. Some similarity was found between percent colonization and PNE using Spearman correlation (rho = 0.544, p < 0.001) There was no correlation between proportion NE and AMF count density (rho = − 0.089, p = 0.362) (Fig. 8).

**Table 5:** Multiple comparison test of AMF colonization along root regions.

Contrast	Percent colonization		Count density		Proportion nutrient exchange	
	Estimate	p-value	Estimate	p-value	Estimate	p-value
MID-BOT	0.009	0.410	0.000032	0.345	0.408	0.011
MID-TOP	− 0.023	0.005	− 0.000077	0.004	− 0.311	0.070
TOP-BOT	0.031	< 0.0001	0.000109	< 0.0001	0.719	< 0.0001

## Discussion

### Preferential AMF colonization in sorghum roots

The 15 cm of roots closest to the soil surface presented highest total AMF colonization and proportion of nutrient exchange fungal structures (Figs. 4 and 6**, **Tables 4 and 5). The total AMF richness and colonization at 0–10 cm soil depth were shown to be higher than at deeper soil layers in *Pedicularis* *kansuensis* at a subalpine grassland ecosystem^62^. Kabir et al. found that total colonization, total hyphae density, and spore density were highest at a depth of 0–15 cm of soil in corn roots. Top roots of sorghum produce secondary roots and fine root via branching. The growing tip is at the bottom of the root. One hypothesis is that the AMF preferentially colonize the actively branching sections of sorghum roots. An alternative hypothesis is that the top layer is rich in phosphorus, defining the niche for AMF colonization^62^. One way to test the first hypothesis is to examine the variation in root morphology between inbred lines with the software DIRT^63^ to see whether or not root morphology has an impact on fungal structure counts. The latter hypothesis could also be tested by using nanodots or phosphorus labeling experiments to track the exchange in the top layer^64^.

### Plant level variance of AMF colonization

In the null model, the total variance in AMF colonization was composed of the sampling variance and plant level variance. Expanding the null models transferred the plant level variance from the variance components to the fungal structure morphology traits. One hypothesis for the result is that sorghum plants of different genotypic backgrounds determine overall AMF abundance in roots by manipulating the developmental stage of AMF species after colonization. Alternatively, in lifestyle studies of single AMF species, researchers found that the composition and morphology of their fungal structures vary. Sorghum lines may match with a community of AMF species in the soil that generates different compositions of AM fungal structures in roots^65^.

Our experimental design does not permit direct testing of the amount of variance in AMF colonization that is under the genomic control of plant hosts, because there were no biological replicates of the RILs. If biological replicates were available, plant level variance would provide an estimate of population level variance of AMF colonization and the measurements, ICCs and VPCs, would capture the broad sense heritability including additive, dominant and epistatic effects. The results in Table 2 are suggestive of performing a large scale Genome Wide Association Study (GWAS) and or Recombinant Inbred Lines (RILs) study in *Sorghum bicolor* to test whether or not sorghum genes play a role in AMF colonization^16,17^.

### Differential AMF colonization between sorghum inbred lines

Percent colonization was a new measurement of overall AMF abundance by taking the ratio of pixelwise segmentation of fungal structures and sorghum roots of our computer vision model. Count density was a similar measurement of AMF abundance to the output of McGonigle^50^ scoring method. The proportion of variance at plant level of these two phenotypes did not lose to the integration of fungal structure morphology traits into the models, which establishes them as favorable response variables for Genome-wide Association Studies of AMF colonization in sorghum populations. While both phenotypes were modeled with fungal structure morphology traits as fixed effects, the fixed effects differed in their ability to explain variability in the data. Two morphological traits of arbuscule were required to explain 95.7% variability in percent colonization suggestive of a mechanism for how the plant controls AMF colonization. One example of a gene relevant to this mechanism encodes the protein DELLA^31,66^.

Count density was modeled with four fungal structures. The final fit to predicting count density explained 87.2% of variability. One could argue that percent colonization is a better phenotype for GWAS for its simplicity, goodness of fit and higher plant level variance. The plant level variance of the phenotype would decrease as the number of plant replicates and sorghum inbred lines grow in scale. Count density should not be discarded as technologies provide newer features to quantify AMF abundance. Finally, the two phenotypes did rank the twelve sorghum inbred lines in different orders. Inbred line L8 had the highest AMF count density but ranked 7th for percent colonization. Ranks for most other inbred lines were comparable. It is recommended to use both phenotypes in future studies of AMF colonization using computer vision.

### Role of models across scales to understand the AMF symbiosis with plants

To understand the symbiosis of *Sorghum bicolor* with AMF it has been necessary here to develop models that operate at multiple scales^67^. First, computer vision models were developed that provided the high throughput data to describe how AMF colonize the plant at the individual plant level. A variety of measures were developed that then can be used in GWAS studies to test the role of the plant genome in shaping the AMF microbiome. To avoid the confounding effects of environmental field variables in a GWAS, it will important to use factorial designs^68^ and blocking to separate the effects of accession from environmental field variables, like Nitrogen and Phosphorus levels. These models operated at the individual level of plants in the study. The computer vision models provided a diverse array of measures to describe the colonization process described above (Tables 2 and 3). We are in the process of not only automating classification and segmentation of fungal structures but full automation of image acquisition as well. A final limitation of the computer vision phenotyping method here is not having live cell images to follow the dynamics of the structures. The Kokkoris laboratory^69^ has been able to follow the dynamics of nuclei in AMF, and it would be useful to adopt a similar approach to other AMF structures to gain a time dimension on colonization. Live cell imaging will also contribute to understanding the prepenetration apparatus^9^, arbuscule formation^31^, and AMF inheritance^12^.

On top of the computer vision model for feature extraction, mixed linear models were added to discover relations between measures of overall AMF abundance with fungal structure morphology and root niche. This mixed linear modeling approach provides a framework for GWAS and QTL mapping by suggesting underlying mechanisms by which the plant genome controls the AMF community. For example, arbuscule count entered into the prediction. This relation suggests a mechanistic link to the arbuscules in how the plant genome controls the AMF microbiome. There are likely genes in both sides of the partnership controlling the development of these structures^70^. Other layers to the modeling will need to be added to conceptualize our understanding of this ancient and fundamental symbiosis^67^.

## Materials and methods

### The Georgia dataset

#### Plant cultivation

Sorghum plants were derived from Recombinant Inbred Lines (RILs), a mapping population consisting of 191 F3:5 from a cross between an unnamed accession of *Sorghum propinquum* (William Rooney, Texas A & M University, College Station, TX) and inbred line TX7000 of *S. bicolo*r^71^. The seeds were kindly provided by Jeff Bennetzen, one of the PIs who constructed the RIL collection^71^. No permissions are needed to use these Sorghum accessions. Three seeds from 15 RILs were planted on October 5, 2020 in steam sterilized Sungro garden soil in 2.5-gallon pots at the UGA Botany Greenhouse. Seedlings were grown on a 11-h light cycle. Plants were fertilized with 1 tablespoon Osmocote. Individual seedling was transferred in 2.5-gallon pots filled with a 4:1 mix of steam sterilized turface and soil from Ironhorse Farm, Watkinsville, GA (Table 1) on day 15. Seedlings were grown to maturity on a 11 h Light/Dark cycle with watering as needed. In addition, one commercial hybrid forage sorghum plant derived from Richardson, TX was harvested at Iron Horse Farm, GA on October 13, 2020, two grain sorghums of accession M72GB7 at Iron Horse Farm, GA on November 12, 2020, a Colby sorghum at UGA Botany Greenhouse. All the methods were carried out in accordance with relevant Institutional guidelines and regulations.

#### Root imaging

Random samples of 0.25 g of fine roots were taken from the whole roots of Richardson, M72GB7, Colby, E46-W, N88, E24, E46 for training images. The cleaned whole roots were cut into 1 cm pieces. Fine roots with intact cortex were randomly selected and weighed to get 3 cassettes of 0.25 g of samples per plant. Root samples were cleared in 10% alkaline hydrogen peroxide solution for 2 h and in 5% KOH overnight at room temperature. Fungal structures were stained using a modified Ink and Vinegar method^72^. Stained roots were spread and flattened on slides prior for imaging. Mounted root samples were imaged at 200× magnification with a Zeiss Primo Star compound microscope equipped with an Axiocam 105 color camera. Focusing was done locally and manually for every field of view during imaging to increase sharpness, but no post image acquisition processing was involved, such as adjusting contrast. McGonigle method was used to generate images at 192 root intersections. The root intersections were 0.5 cm equidistantly spread across a 75 × 25 mm glass slide. The fungal structures at root intersections were manually scored and annotated for training the computer vision model.

To test for the differential AMF colonization in root regions and in sorghum plants, 12 RIL sorghums of E37, L8, N6-F3, N10, N43, N66, N68, N102, N108, N110, N116, and N162 were sampled from three root regions. The ‘TOP’ region was the first 15 cm of roots below soil surface. The ‘MID’ region is the next 15 cm below. The ‘BOT’ region was roots longer than 30 cm (Fig. 4). Aerial roots were excluded from sampling. From each region of a plant, 3 technical replicates of 0.25 g of fine roots with intact cortex were randomly sampled. Each plant was represented with a total of 9 cassettes or 2.25 g of root samples. A line has only 1 plant as biological replicate. The same clearing, staining and imaging procedures were applied.

### The Cambridge dataset

The publicly available Cambridge dataset (zenodo ID 10.5281/zenodo.5118948) included 15 whole slide scanning images acquired with an Epson Perfection flatbed scanner (Epson UK, Hemel Hempstead, UK) using default settings and a resolution of 3200 dots per inch. The images were downloaded from the zenodo dataportal^73^ using the zenodo-get software method. The 15 whole slide images were in jpg format and 10,389 × 5108 pixels and 96 pixels/inch in size. The original annotations were discarded. The same annotators and annotation standards for the Georgia dataset were used in reannotation to maintain uniformity. The annotated images were tiled and added to the Georgia dataset to create a secondary training set for more and even representation of each AMF class.

### Image annotation

The root image annotation was conducted using the VGG annotator tool^74^. The fungal and root structures were manually annotated using the polygon tool. One of seven class labels was assigned to a structure (Table 1). The annotation results were exported as a json file and csv table.

All 746 jpg images in the Georgia dataset were segmented and annotated. We generated 3577 polygon annotations. A total of 14 out of 15 images were selected from the Cambridge dataset and produced 20,588 annotations.

**Table:** 

Class	Annotation rules for the Georgia dataset
Root	Plant root
Extradical hypha (exH)	Filamentous structure outside the boundary of a plant root annotation
Intraradical hypha (inH)	Filamentous structure within the boundary of a plant root annotation
Spore (sp)	Circular structures with a solid outline, connected to AMF external hypha, and outside the boundary of plant root
Vesicle (ves)	Circular or rectangular structures with a solid outline and within the boundary of plant root
Arbuscule (arb)	Highly branched hypha with fuzzy outline and connected to intraradical hyphae within the boundary of plant root
Others	Non-AM fungal structures

### Data cleaning

The Georgia and the Cambridge datasets were cleaned to produce similar input data. All segmentation shapes were approximated by polygons, including converting polyline to polygon directly and resampling points in circles to produce polygons. Some segmentation shapes, including point, rectangle and ellipse, were removed. Empty and undefined segmentations were also removed. Classes with few representative examples were merged into ‘others’. Class labels were made uniform in their vocabularies. The final class list included root, AMF internal hypha, AMF external hypha, AMF arbuscule, AMF vesicle, AMF spore and others. The cleaned Georgia dataset included 746 jpg images that are 2380 × 1740 pixels and 300 pixels/inch in size and 3577 annotations.

The Cambridge dataset needed additional processing steps. To have comparable input data in size, the 14 images were tiled and subsampled. The images were tiled into squares of 512 × 512 pixels and smaller images on the boundaries. The segmentations were subsampled to fit each tile. New segmentation polygons were produced at the intersection of the tiles and the original segmentations using Shapely^75^. Polygons with self-intersection were dissected into smaller simple polygons. Points and LineStrings were ignored as subsampling results. Indices for segmentation and bounding box were recalculated relative to the new tiled image. Only tiled images with at least one segmentation annotation were kept. The quality of tiling and subsampling were checked by comparing segmentation in the raw images and the tiled small images visually. The resulting Cambridge dataset included 1379 tiled jpg images that are 512 × 512 pixels and 96 pixels/inch in size and 20,558 annotations.

The Georgia dataset were separated into training, validation and testing sets at 8:1:1 ratio. The training set has 598 images and 2874 annotations. To increase the number of examples for each fungal structure, the Cambridge dataset was divided in the same 8:1:1 ratio and merged to the previous Georgia training, validation and testing sets. The secondary training set is made up of 1105 images and 16,417 annotations.

The final prediction set was consisted of 24,391 root images from the ‘TOP’, ‘MID’, and ‘BOT’ regions of 12 sorghum RIL plants. Images with height or width less than 100 pixels in the prediction set were dropped.

### Mask R-CNN model training

Mask R-CNN was implemented in Detectron2^46,76^ and is composed of the backbone, the region proposal network (RPN), and heads^46,77^. The ResNet 50 and FPN (Feature Pyramid Network) backbone extracts feature map from images^46,78^. RPN proposes candidate regions^79^. Heads produce bounding box, mask, and class inferences. The Mask R-CNN model was pretrained on the COCO dataset with 3 × schedule^46,76,80^. The pretrained model was retrained on the first and secondary training sets for 50 epochs with batch size 2 and the default learning rate schedule.

Different hyperparameters were tested, and each combination was repeated three times with different random seeds. Learning rates of 0.001 and 0.002 were tested. The number of frozen or fine-tuned backbone modules was varied by changing the ‘FREEZE_AT’ parameter from 1 to 3. Two augmentation options were implemented. The default option included image random flip and resize, and the second option added random crop, rotation, and brightness adjustment as augmentation options. Other parameters were set to the defaults in Detectron2 configuration^76^.

Model performance and hyperparameters were evaluated based on mean Average Precision (mAP). The best fine-tuned model for defined hyperparameters was selected based on total loss in validation set during training^46^.

The model quality metric mAP was calculated with varying confidence thresholds and averaged over all classes. In addition, AP50 was calculated at Intersection over union (IoU) level 50%, and AP was averaged over IoU levels from 50 to 95%. Score threshold for inference in test set was set to 0.7.

### Mixed linear model prediction and statistical analysis

The best model was used for the prediction set of 24,391 images. Other settings remained the same as training. Inferred segmentations in an image were cross-tabulated by class versus segmentation number and pixel number. For downstream statistical analysis on AMF colonization, three class level statistics were generated using the two outputs above.

Count *density* of an AMF structure was defined as its segmentation number divided by the root pixel number (count/pixel). *Average class size* of an AMF structure was its pixel number divided by its segmentation number (pixel/count). *Percent colonization* by an AMF structure was measured as its pixel number per root pixel (pixel/pixel). The three class level colonization statistics were calculated for every slide. A total of 648 entries was used for regression analysis to test for differential colonization in root regions and sorghum plants.

Mixed effect models in ‘lme4’ R package^81^ were used for modeling the three class level AMF colonization statistics. ANOVA and t-tests were used to test for the significance of model parameters. Likelihood ratio test was used to test the significance of a model to a nested model.

### Computational resources

Model training and inference was implemented on sapelo2 at the Georgia Advanced Computing Resource Center (GACRC) with one p100 GPU, 4 CPUs, and 20 GB memory. GPUs were used for model training. CPUs were used for model inference. Codes are available in GitHub: https://github.com/Arnold-Lab/image_seg_sorghum_am.

## Data Availability

Summary data and codes are available in GitHub: https://github.com/Arnold-Lab/image_seg_sorghum_am. The analyses and manuscript are available in RStudio with the exception of the tables, which were converted manually back to Word formatting from image formatting at the request of the publisher. The large collection of over 20,000 raw images are available upon reasonable request from a shared DropBox folder.
